# Influence of adjunctive azithromycin on microbiological and clinical outcomes in periodontitis patients: 6-month results of randomized controlled clinical trial

**DOI:** 10.1186/s12903-020-01209-0

**Published:** 2020-09-01

**Authors:** Katarina Čuk, Katja Povšič, Suzana Milavec, Katja Seme, Rok Gašperšič

**Affiliations:** 1 Department of Oral Medicine and Periodontology, Faculty of Medicine, University of Ljubljana, Hrvatski trg 6, 1000 Ljubljana, Slovenia; 2 Nova Gorica Health Centre, 5000 Nova Gorica, Slovenia; 3 Institute of Microbiology and Immunology, Faculty of Medicine, University of Ljubljana, 1000 Ljubljana, Slovenia

**Keywords:** Periodontitis, Azithromycin, Scaling and root planing, Microbiology

## Abstract

**Background:**

Our aim was to determine if azithromycin therapy, as an adjunct to scaling and root planing (SRP), decreases the number of pathobiontic subgingival plaque species and sites demonstrating pocket depth (PD) ≥ 5 mm and bleeding on probing (BOP) 6 months post-treatment.

**Methods:**

In a double-blind randomized parallel-arm placebo-controlled trial, 40 patients received nonsurgical periodontal treatment in two sessions within 7 days. Patients then received systemic antibiotic therapy (*n* = 20, azithromycin 500 mg/day for 3 days) or placebo (n = 20). Pooled microbiologic samples were taken before and 6 months after therapy and analysed by established culture methods. The primary outcome variable was the number of sites with PD ≥ 5 mm and BOP at the 6-month re-evaluation. Using multivariate multilevel logistic regression, the effects of gender, age, antibiotic therapy, presence of *P. gingivalis* or *A. actinomycetemcomitans*, smoking, tooth being a molar and interdental location were evaluated.

**Results:**

The number of sites with PD ≥ 5 mm and BOP after 6 months was similar in the test (Me = 4, IQR = 0–11) and control (Me = 5, IQR = 1–22) group. Adjunctive azithromycin treatment, compared to SRP alone, resulted in more frequent eradication of *A. actinomycetemcomitans* (*p* = 0.013) and *C. rectus* (*p* = 0.029), decreased proportion (*p* = 0.006) and total counts (*p* = 0.003) of *P. gingivalis*, and decreased proportion of *C. rectus* (*p* = 0.012). Both groups showed substantial but equivalent improvements in periodontal parameters, with no intergroups differences at initially shallow or deep sites. The logistic regression showed a lower odds ratio for healing of diseased sites on molars (OR = 0.51; *p* <  0,001).

**Conclusion:**

Despite significant changes in numbers of *A. actinomycetemcomitans*, *P. gingivalis* and *C. rectus*, patients with periodontitis do not benefit from adjunctive systemic azithromycin in terms of number of persisting sites with PD ≥ 5 mm and BOP.

**Trial registration:**

EUDRA-CT: 2015–004306-42; https://www.clinicaltrialsregister.eu/ctr-search/trial/2015-004306-42/SI, registered 17. 12. 2015.

## Background

Periodontal treatment of the individual patient is a series of four sequential parts: the systemic, initial, corrective and maintenance phases [1]. A critical point arises after the initial phase, when the success of mechanical debridement is evaluated in terms of sites with probing depth (PD) ≥ 5 mm and bleeding on probing (BOP), and a decision regarding the need for surgical intervention has to be made [2, 3]. Regardless of the chosen protocol, mechanical treatment is essential for the disruption and removal of subgingival biofilms, but the effects may be enhanced by other treatment modalities including therapy with antibiotics [4].

Amidst the flood of all available antimicrobials, the empiric prescription of systemic amoxicillin/metronidazole, as an adjunct to scaling and root planing (SRP), remains the gold standard [5]. Alternatively, it has often been suggested that azithromycin can be used as a second-choice antibiotic [5, 6], yet an equivalence to clinical results obtained with amoxicillin/metronidazole treatment has not been demonstrated to date [7]. Its advantages include rare side effects [8], favourable pharmacological properties [9] and a short dosage regimen due to its long half-life [10], resulting in good patient compliance [11]. Its three main modes of action consist of bacteriostatic activity, anti-inflammatory action and extended release as a result of persistence in fibroblasts and leukocytes that reside/migrate into periodontal tissues [12].

The outcomes of studies assessing the benefits of azithromycin remain contradictory. Several of them lasting from 3 to 12 months and administering from 500 to 2000 mg of azithromycin/day in time frames of 3–7 days, have shown not only improvements in inflammatory, biochemical and clinical parameters, but also significant changes in the composition of subgingival biofilms, often resulting in the eradication of *P. gingivalis* [11, 13–19]. Azithromycin has shown favourable results in the treatment of both aggressive [20] and mild to moderate chronic periodontitis [13, 15] and has also shown promising results in smokers [21]. According to systematic reviews [22, 23], the additional clinical benefits of azithromycin are most pronounced in deeper pockets (≥6 mm). A recent meta-analysis [5] also concluded that the benefit of azithromycin in reducing the PD of initially deep pockets can be observed after 1 year. The evidence of azithromycin was, nevertheless, evaluated as moderate compared to the more consistently found benefits of amoxicillin/metronidazole treatment. It has therefore been suggested that the use of azithromycin should be limited to subjects who would receive the maximum benefit from these agents, i.e. subjects with more severe periodontal destruction [24].

On the contrary, the outcomes of several clinical trials focused on severe periodontitis cases did not indicate any significant differences in clinical and/or microbiologic parameters between subjects treated with azithromycin compared to those treated with placebo [18, 25]. Even though some of these studies reported a significant decrease in the proportion and/or total counts of pathobiontic species, these changes were not reflected in the clinical parameters 6 months post treatment and did not show any significant improvements in periodontal parameters, when compared to subjects who were treated with SRP alone [25]. Thus, the notion of Zhang et at [24]., stating that decreases in mean counts of pathobiontic species are significantly associated with improvements in attachment loss and reductions of PD, may be questioned.

Surgical intervention is indicated if pockets measuring ≥5 mm [2, 26] or ≥ 6 mm [27] persist after initial treatment due to enhanced risk for disease recurrence and tooth loss. When antibiotics are used, pocket closure is more likely to occur 6 months than 3 months post-treatment [27]. Since small changes in microbiological and clinical parameters do not automatically change the treatment regimen (the need for surgical intervention after nonsurgical treatment phase), the clinical significance of studies which demonstrated small but statistically significant improvements in microbiological (decreased numbers of pathobiontic species) and clinical parameters (PD/ clinical attachment loss) after adjunct systemic azithromycin treatment is questionable. In addition, most of these studies completely neglect the intra-individual variabilities of observed parameters (i.e. variability between different sites and teeth), thereby showing a tendency towards false positive interpretation. Our aim was to overcome these shortcomings by defining the primary outcome as a distinct entity – the number of sites with probing depth (PD) ≥ 5 mm and bleeding on probing (BOP), usually perceived to require additional surgical treatment after mechanical debridement [2, 3, 26, 27] – and creating a multilevel statistical model, thus also taking into account the nesting of data within teeth and patients. Our null hypothesis assumed that the number of diseased sites (DS) with probing depth (PD) ≥ 5 mm and bleeding on probing (BOP) would not be lower 6 months after adjunct azithromycin administration than after conventional non-surgical periodontal treatment. The prognostic value of additional systemic azithromycin therapy for the healing of residual DS was additionally evaluated in a multivariate multilevel regression model accounting for the influence of microbiological and other risk factors.

## Methods

The protocol of the present 6 month, single-centre, randomized, placebo-controlled, parallel-design, double blind clinical trial was approved by the National Medical Ethic Committee (46/08/15) and the Agency for Medicinal Products and Medical Devices of the Republic of Slovenia. It was also registered at the EU Clinical Trial Register (EUDRA-CT: 2015–004306-42). Written informed consent forms were collected from all subjects before participation. The study was performed in line with the principles of the Declaration of Helsinki.

### Study population

40 out of 732 consecutively evaluated individuals referred to the Department of Oral Medicine and Periodontology, University Dental Clinic of Ljubljana, Slovenia, for periodontal treatment between March 2016 and March 2018 were carefully selected as participants in the study. The inclusion criteria were: age 25 to 70 years, untreated moderate to advanced periodontitis with probing depth (PD) ≥ 5 mm at a minimum of four teeth in four different quadrants (stage III or IV according to the AAP/EFP classification of 2018), absence of systemic disease or medication, presence of at least 20 teeth (excluding third molars) ensuring a stable occlusion. The exclusion criteria were: treatment with systemic antibiotics in the past 12 months, suspected or confirmed intolerance to azithromycin, any kind of periodontal therapy in the past 12 months, presence of implants, fixed and/or removable prosthetic restorations, pregnancy or lactation, and systemic disease influencing periodontal tissues, healing processes or immune functions. Smokers were not excluded from the study.

### Clinical protocol

The same clinical protocol as described in a previously published article was followed [28]. In brief: at baseline, an experienced, calibrated examiner (R. G.) conducted a full periodontal examination of each patient using a manual Williams probe (POW6, Hu-Friedy, Chicago, Illinois, USA). The recorded clinical variables were: presence/absence of bleeding upon gentle probing around the gingival crevice using a dichotomous gingival bleeding index (GBI), presence/absence of plaque deposits using a dichotomous plaque index (PlI), PD, presence/absence of BOP, gingival recession (REC). All of the described parameters were assessed at 6 sites on each tooth. Clinical attachment loss (CAL) was calculated as a sum of PD and REC. Since surgical intervention is indicated for pockets measuring ≥5 mm and further clinical attachment loss is expected at sites that demonstrate persistent BOP, sites demonstrating both features (PD ≥ 5 mm + BOP) were defined as diseased sites (DS); the number of DS per patient was calculated [2, 3, 28]. Furcation involvement and tooth mobility was assessed for each tooth but not included in the report.

After the baseline examination, each of the 40 subjects underwent non-surgical periodontal therapy encompassing: motivation and instruction in proper oral hygiene, the removal of supra- and subgingival deposits using piezoelectric ultrasonic instruments (PiezoLED ultrasonic scaler with Piezo Scaler tip 203 (KaVo dental, Biberach, Germany)), followed by scaling and root planing of sites with PD ≥ 5 mm under local anaesthesia (Ultracain©, Hoechst, France) using Gracey curettes (Hu-Friedy, USA). All patients received two 1.5-h sessions of non-surgical therapy, performed by the same operator (S. M.) within 7 days. Oral hygiene instructions were reinforced at each visit.

At the end of the second session, each subject received a box containing 3 film coated tablets and was instructed to take one per day for 3 consecutive days. All tablets and boxes were identical in appearance; the boxes were only marked with the sequential patient number. Whether the box contained test medication (Azibiot 500 mg; 20 subjects) or placebo (20 subjects) was determined using a computer-generated randomisation table prepared by Krka d. d. (Novo Mesto, Slovenia) and disclosed to the examiner and operator after the last follow-up visit of the last patient, ensuring that all measurements were performed by a blinded investigator.

Seven days after treatment, the first follow up visit was scheduled. Subjects were asked if they had taken the remaining two prescribed pills and were required to return the packaging of the medication for compliance control. Reports of any adverse effects were noted. Subjects were then recalled 3 and 6 months after treatment. At each appointment, a full mouth periodontal examination following the same protocol as at baseline and using the same type of periodontal probe (POW6, Hu-Friedy, Chicago, Illinois, USA) was done by the same blinded examiner (R. G.). At the 3-month follow up visit, all sites with PD ≥ 5 mm and BOP were re-instrumented using the PiezoLED ultrasonic scaler with Piezo Scaler tip 203 (KaVo dental, Biberach, Germany). Oral hygiene instructions were also reinforced.

### Microbiological sampling

At baseline, pooled subgingival microbiologic samples were taken from each individual at 4 deepest PD sites of each jaw quadrant with two absorbent paper points (diameter: 0.30 mm; Maillefer, Ballaigues, Switzerland). All samples of an individual subject were placed into the same test tube containing 1.5 mL of reduced transport fluid. Within 4 h, samples were analysed at the Institute of Microbiology, Faculty of Medicine, University of Ljubljana, Slovenia, by established anaerobic culture methods. After 7 days, the number of colonies of *Capnocytophaga ochracea, Campylobacter rectus, Eikenella corrodens, Fusobacterium nucleatum, Parvimonas micra* and *Prevotella intermedia* were counted. The number of colonies of *Porphyromonas gingivalis* and *Tannerella forsythia* were counted after 14 days. Serially diluted samples were also plated onto Dentaid-1 medium for a 3-day incubation period in air with 5% CO_2_ at 37 °C, allowing for the evaluation of *Aggregatibacter actinomycetemcomitans* colonies after 3–5 days. Bacterial colonies were identified using matrix-assisted laser desorption/ionization time-of-flight mass-spectrometry (MALDI TOF MS) (MBT COMPASS 4.1, Microflex, Bruker Daltonics, Bremen, Germany) on plates with total colony counts of 30–300 colonies. The numbers of colonies pertaining to each of the bacterial species under investigation were counted and the number of colonies per 1 mL of sample (colony forming units, CFU/mL) was calculated. Proportions of individual colonies were calculated relative to the total number of anaerobic bacteria colonies on every plate. Protocol adjustments and calibrations were performed with the reference laboratory before microbiological analysis [29, 30].

### Statistical analysis

Since sites with PD ≥ 5 mm and BOP are commonly perceived to require surgical treatment in addition to non-surgical therapy, the primary outcome measure of this study was the number of diseased sites per subject (DS), defined as the number of sites with PD ≥ 5 mm and BOP. A sample size that would allow for an intergroup difference of 2 DS, a standard deviation of 2 DS, 90% statistical power and statistical significance at *p* <  0.05 was calculated to be 16 subjects per group. Sample size was, however, increased by 15% since the collected data was not normally distributed and non-parametric statistical tests had to be used. The secondary outcome measures included differences between the test and control groups for 6-month post-treatment values and changes in PD, REC, CAL, BOP, the number of molars and interdental spaces with DS, the number of healed sites (HS) as well as differences in the prevalence, proportion and total counts of colony forming units (CFU/mL) under observation.

Baseline intergroup differences were compared by t-test (age), Fisher’s exact probability test (smoking, gender) and Wilcoxon’s rank sum test (clinical and microbiological parameters). Intragroup baseline and 6-month post-treatment clinical and microbiological parameters were compared by Wilcoxon’s signed rank test. The Chi square test (intergroup changes) and McNemar’s test (intragroup changes between baseline and 6-month follow-up measurements) were used to evaluate differences in the prevalence of bacterial species. As no corrections were made for multiple comparisons, adjustments of control variables were made using a multilevel multivariate regression model, which took into account the nesting of sites within teeth and the nesting of teeth within patients. The healing of DS was determined as a binary dependent variable. The random intercept was individually predicted for every subject and tooth, allowing for variability in the probability of site healing with regards to each tooth and subject. The multivariate multilevel logistic regression model was used to account for the interdependent influence of gender, age, smoking, tooth being a molar, interdental site location, treatment mode and the presence/absence of specific bacteria at baseline on the healing of diseased sites 6 months after treatment [28]. SPSS v. 26 was used for the analyses. The significance threshold was set at α = 0.05.

## Results

**Fig. 1:**
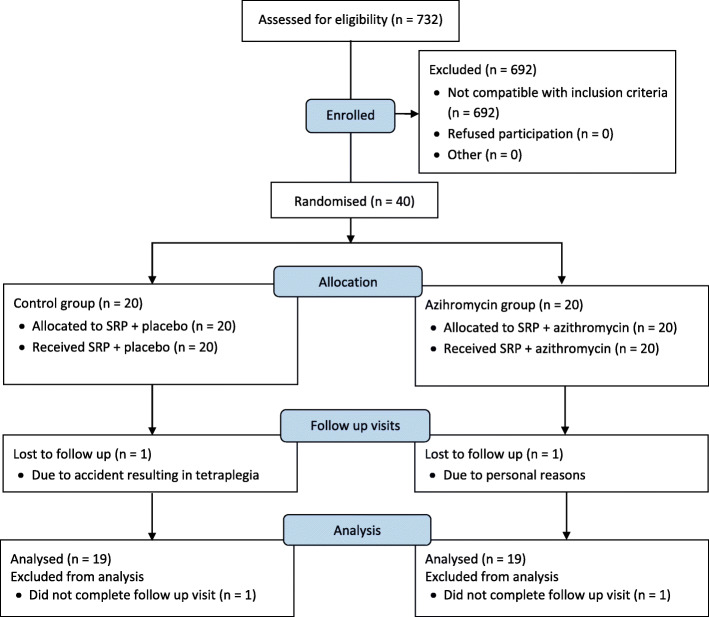
CONSORT flow diagram

In total, 40 subjects received initial microbiological screening and baseline treatment. Two patients, one from each treatment group, dropped out due to personal reasons and were excluded from the study (Fig. 1). The statistical analysis thus included 38 subjects, 19 in the control and 19 in the test group, with a mean age of 45.4 (SD = 10.5) and 44.0 (SD = 8.5) years, respectively (*p* = 0.650). The control group consisted of 14 males while the test group consisted of 12 males (*p* = 1.000). Five smokers were present in the control and 4 in the test group (p = 1.000).

A small amount of adverse effects was reported in both groups. One subject in the test group experienced nausea, while one subject in the control and two in the test group reported having headaches, probably associated with the study medication.

### Clinical results

A total of 495 teeth in the test and 485 teeth in the control group were analysed. The mean number of teeth in both groups was 26 (the SD of the test and control groups was 1.5 and 2.4, respectively; *p* = 0.732). The median number of DS at baseline was 47 in the control group and 34 in the test group; these decreased to 5 and 4 at the 6-month follow-up visit, respectively. In control group subjects, a median of 15 DS were found on molars at baseline and only 1 at the 6-month follow-up, while test group subjects harboured a median of 18 DS on molars at baseline and 2 at the 6-month follow-up. At baseline, the median number of periodontal pockets deeper than 6 mm was 15 in the test and 14 in the control group. This number decreased to 1 in both groups 6 months after treatment. Median values and interquartile ranges (IQR) of the included clinical variables at baseline and 6 months post-treatment are shown in Table 1. Clinical measurements at baseline were comparable to those found at the 6-month check-up. Statistically significant intergroup differences were thus not found, while statistically significant intragroup differences (baseline vs. 6-month clinical characteristics) were observed in both the control and test group.

**Table 1:** Baseline and 6-month characteristics of treatment groups (Me [IQR])

	Baseline characteristics			6-month characteristics		
	Control group	Test group	p	Control group	Test group	p
Teeth with DS (n)	18.0 (16.3–21.3)	14.0 (12.3–22.3)	0.412	4.0^a^ (1.0–10.0)	4.0^a^ (0.0–7.0)	0.616
DS (n)	47.0 (28.0–63.8)	34.0 (23.3–62.3)	0.397	5.0^a^ (1.0–22.3)	4.0^a^ (0.0–11.0)	0.497
DS on molars (n)	15.0 (10.3–23.0)	18.0 (11.3–23.5)	0.661	1.0^a^ (0.3–6.8)	2.0^a^ (0.0–6.5)	0.801
PlI (%)	40.0 (20.0–50.0)	30.0 (20.0–50.0)	0.726	10.0^a^ (2.0–20.0)	10.0^a^ (4.0–10.0)	0.988
PD (mm)	4.1 (3.6–4.5)	3.9 (3.7–4.5)	0.838	2.9^a^ (2.4–3.6)	2.7^a^ (2.4–3.1)	0.484
REC (mm)	0.4 (0.3–0.6)	0.4 (0.2–0.7)	0.895	0.7^a^ (0.5–1.1)	0.6^a^ (0.5–1.0)	0.661
CAL (mm)	4.2 (3.6–4.9)	4.2 (3.7–4.8)	0.988	3.3^a^ (2.6–4.3)	3.3^a^ (2.7–3.7)	0.770
BOP (%)	80.0 (60.0–90.0)	70.0 (50.0–90.0)	0.620	20.0^a^ (10.0–40.0)	20.0^a^ (10.0–20.0)	0.651

*Me* median value, *IQR* interquartile range, *DS* diseased sites (PD ≥ 5 mm + BOP); *PlI* plaque index; *PD* probing depth; *REC* recession; *CAL* clinical attachment loss; *BOP* bleeding on probing; ^a^, statistically significant change in comparison to baseline

Both groups showed improvements in all periodontal parameters 6 months after treatment. Even though greater reductions in PD, BOP and CAL occurred in the test group and greater reductions in GBI and PlI occurred in the control group, no statistically significant intergroup differences were found – this applies for all measuring sites and separately for initially DS with PD ≥ 5 mm and BOP, initially deep probing sites (PD ≥ 5 mm) without BOP and initially shallow pockets (PD < 5 mm). BOP was similar in both groups (Table 2).

**Table 2:** Differences in periodontal parameters between baseline and 6-month follow-up visit (Me [IQR])

	Control group	Test group	p
**All probing sites**			
PlI (%)	29.0 (15.2–32.6)	22.6 (14.8–34.1)	0.589
GBI (%)	7.6 (0.0–26.2)	3.7 (1.9–10.2)	0.529
PD (mm)	1.1 (0.8–1.6)	1.4 (1.1–1.5)	0.358
REC (mm)	− 0.2 (− 0.6 – − 0.1)	− 0.3 (− 0.4 – − 0.1)	0.672
CAL (mm)	0.7 (0.5–1.2)	1.0 (0.7–1.3)	0.287
BOP (%)	51.2 (36.0–64.4)	49.4 (31.1–66.7)	0.930
**Sites with initial PD < 5 mm**			
PlI (%)	20.2 (15.1–30.9)	16.1 (9.8–31.0)	0.726
GBI (%)	8.3 (0.0–22.5)	3.3 (0.6–9.1)	0.547
PD (mm)	0.5 (0.3–0.7)	0.7 (0.5–0.8)	0.068
REC (mm)	− 0.2 (− 0.5 – − 0.1)	−0.2 (− 0.3–0.0)	0.511
CAL (mm)	0.3 (− 0.1–0.6)	0.5 (0.2–0.7)	0.140
BOP (%)	46.9 (37.6–63.7)	47.1 (29.7–65.3)	0.884
**Initially deep probing sites (PD ≥ 5 mm) without BOP**			
PlI (%)	4.5 (0.0–31.8)	27.3 (0.0–35.4)	0.753
GBI (%)	0.0 (0.0–0.0)	0.0 (− 3.3–0.0)	0.601
PD (mm)	2.0 (2.0–2.6)	2.6 (2.2–2.9)	0.110
REC (mm)	−0.6 (− 0.9–0.0)	−0.3 (− 0.5–0.0)	0.274
CAL (mm)	1.9 (1.0–2.3)	2.3 (2.0–2.5)	0.151
BOP (%)	/	/	/
**Initially diseased probing sites (PD ≥ 5 mm and BOP)**			
PlI (%)	31.0 (23.4–47.0)	33.3 (22.2–46.9)	0.804
GBI (%)	7.9 (0.0–30.8)	1.6 (0.0–14.8)	0.486
PD (mm)	2.6 (2.2–2.9)	2.5 (2.3–3.1)	0.849
REC (mm)	−0.4 (− 0.6–0.2)	−0.5 (− 0.6 – − 0.3)	0.693
CAL (mm)	2.1 (1.6–2.4)	2.2 (1.5–2.7)	0.942
BOP (%)	/	/	/

*Me* median value, *IQR* interquartile range, *PlI* plaque index; *GBI* gingival bleeding index; *PD* probing depth; *REC* recession; *CAL* clinical attachment loss; *BOP* bleeding on probing

Roughly 90% of DS (BOP ≥5 mm and BOP) became healed (BOP < 5 mm and/or no BOP) regardless of treatment type and probing site specification (non-molar/molar tooth type and buccal or oral/interdental location) (Supplemental Table 1). The multivariate multilevel logistic regression model showed that site healing was negatively associated with molars when controlling for the rest of the variables (Table 3). Probing sites were found to have a statistically significant (*p* < 0.001) lower odds ratio (OR) for becoming healed if present on molars compared to probing sites on non-molars (OR: 0.51–95% CI: 0.35–0.72). No other variable was identified as a prognostic factor for the healing of initially DS – only smoking showed a tendency towards inferior healing (*p* = 0.099). The 6-month OR for the recovery of test group subjects who were treated with azithromycin did not statistically significantly differ from that of control group subjects (*p* = 0.719).

**Table 3:** Associations between risk factors, probing site locations, treatment types, the presence of periodontopathogens and site healing

	OR (95% CI)	p
Gender (male)	1.31 (0.45–3.84)	0.625
Age	1 (0.94–1.05)	0.893
Smoking	0.39 (0.13–1.20)	0.099
Test group	1.20 (0.44–3.27)	0.719
Molars	0.51 (0.35–0.72)	< 0.001*
Interdental site	0.96 (0.65–1.41)	0.826
*Aggregatibacter actinomycetemcomitans*	1.68 (0.61–4.63)	0.318
*Porphyromonas gingivalis*	0.49 (0.17–1.41)	0.184
*Prevotella intermedia*	0.60 (0.09–4.16)	0.602
*Tannerella forsythia*	0.80 (0.20–3.15)	0.755

*OR* odds ratio, *CI* confidence interval – *, statistically significant change

### Microbiological results

**Fig. 2:**
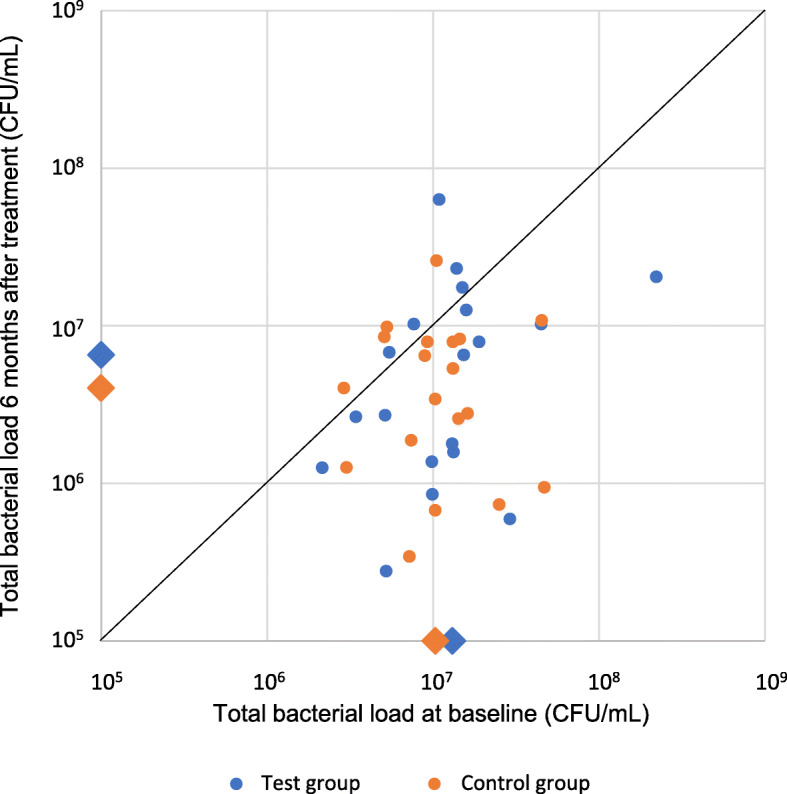
Total bacterial loads at baseline and 6 months after treatment

The baseline median (Me [IQR]) total count of bacteria in the subgingival samples of all 38 subjects together statistically significantly decreased (*p* = 0.001) 6 months after treatment [from 1.07 × 10^7^ (7.24 × 10^6^ to 1.52 × 10^7^) bacteria/ml to 4.69 × 10^6^ (1.42 × 10^6^ to 9.50 × 10^6^)]. Both the test [from 1.31 × 10^7^ (6.55 × 10^6^ to 1.56 × 10^7^)/ml at baseline to 6.53 × 10^6^ (1.48 × 10^6^ to 1.14 × 10^7^)/ml 6 months post-treatment; (*p* = 0.038)] and control [from 1.03 × 10^7^ (7.29 × 10^6^ to 1.44 × 10^7^)/ml at baseline to 4.03 × 10^6^ (1.57 × 10^6^ to 8.09 × 10^6^)/ml 6 months post-treatment; (*p* = 0.007)] group experienced statistically significant intragroup differences in bacterial total counts, while intergroup differences were not observed (Fig. 2).

**Fig. 3:**
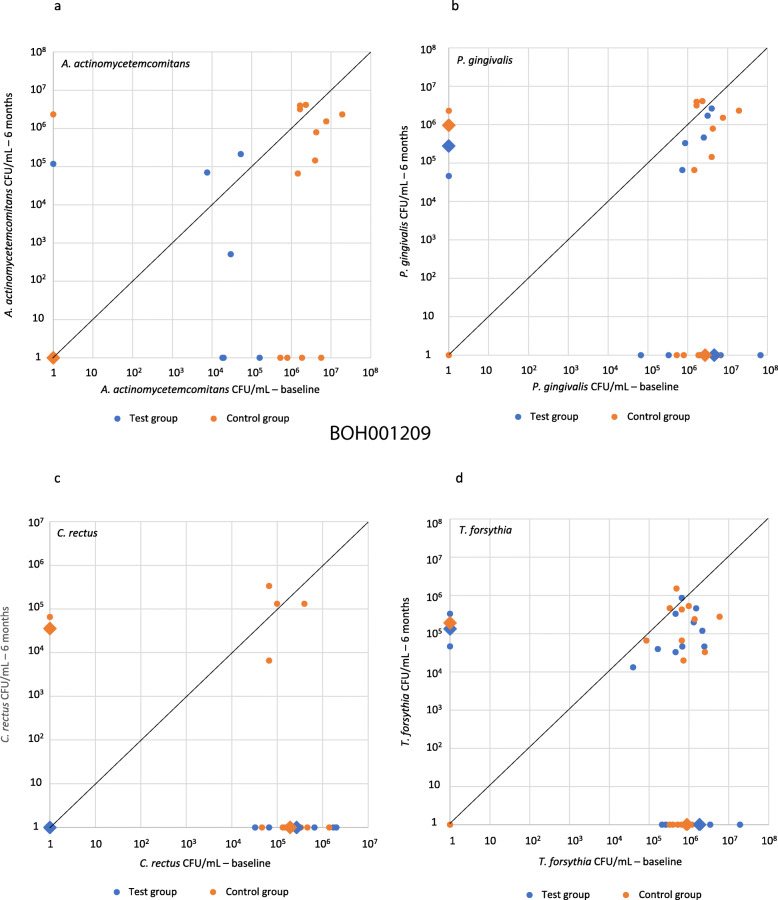
Scatter plots of total counts for *A. actinomycetemcomitans*, *P. gingivalis, C. rectus* and *T. forsythia* at baseline and 6 months after treatment

At baseline, no statistically significant intergroup differences existed with regards to the frequencies of detection, proportions and total counts of the cultivable species under investigation. The test group showed statistically significant decreases in the prevalence of *C. rectus* (*p* = 0.013) and the proportion of *P. gingivalis* (*p* = 0.006), *C. rectus* (*p* = 0.012) and *T. forsythia* (*p* = 0.001), as well as reductions in the total counts of *P. intermedia* (*p* = 0.050), *C. rectus* (p = 0.012), *T. forsythia* (p = 0.001) and *P. gingivalis* (*p* = 0.003) 6 months after treatment, when compared to baseline. The control group demonstrated statistically significant decreases in the prevalence of *C. rectus* (*p* = 0.039), the proportion of *T. forsythia* (*p* = 0.008) and the total counts of *P. intermedia* (*p* = 0.031), *C. rectus* (*p* = 0.033) and *T. forsythia* (p = 0.008) 6 months after treatment, as compared to baseline. No bacterial species under investigation showed overgrowth to indicate super-infection at the 6-month follow-up visit (Fig. 3 and Supplemental Tables 2, 3, 4 and 5).

The 6-month intergroup comparison revealed a statistically significant (*p* = 0.046) lower frequency of detection for *C. rectus*, a more frequent eradication of *A. actinomycetemcomitans* (*p* = 0.013) and *C. rectus* (*p* = 0.029) and lower total counts of *C. rectus* (*p* = 0.018) in the test group.

## Discussion

The primary aim of this study was to determine whether after 6 months the use of azithromycin as an adjunct to scaling and root planing, in comparison to placebo, decreases the number of residual diseased sites, i.e. sites with probing depth (PD) ≥ 5 mm and bleeding on probing (BOP), which are generally considered to require further therapy [2, 3, 26, 27]. It found that azithromycin provided no such benefit. Furthermore, it showed no statistically significant differences as to placebo in the number of healed sites and in the reductions of PD, REC, CAL, GBI and BOP (secondary outcomes). Adjunct azithromycin use did, however, change the microbiological composition of subgingival plaque samples, seen as a reduction in the proportions and total counts of *P. gingivalis,* the proportion of *C. rectus* and the eradication of *A. actinomycetemcomitans* and *C. rectus.* Nevertheless, the microbiologic changes after 6 months were not reflected in the clinical parameters, did not change the protocol of treatment with regards to persisting diseased sites and thus do not support the prescription of azithromycin in cases of stage III/IV periodontitis. It is possible, though, that the clinical outcomes could show greater improvements if monitored for a longer time period due to slower recolonization of shallow periodontal pockets, resulting in the need for a less rigorous maintenance protocol [5].

During the microbiologic analysis, all 9 periodontopathogens were seldomly found in the same subject, resulting in a relatively large number of ‘negative samples’ for specific bacteria. Considering the amount of ‘negative samples’, it should also be noted that the limit of detection using classical microbiologic culture is 10^3^–10^4^ cells/ml, while methods based on DNA fingerprinting have a detection limit of 25–100 cells/ml. A ‘negative sample’ therefore does not necessarily mean that cells of a specific bacterial type were not present, but merely that they might not have been detected, making the number of false negative samples relatively high. On the other hand, small amounts of pathobiontic species, as detected by DNA methods, can also be present in subjects with healthy periodontal tissues and do not have a clinically significant harmful effect [31, 32]. Microbiological methods with higher detection limits may therefore be more appropriate for routine clinical use since they do not identify small amounts of bacteria, compatible with periodontal health. Even though ‘negative samples’ could also be attributed to the variability of sampled pocket depths – biofilm structures between shallow and deep pockets are known to differ greatly [32] – this is not likely since microbiologic samples were taken from the deepest probing site of each examined quadrant, always exceeding PD of 6 mm. In addition, protocol adjustments were made with the reference laboratory before microbiological analysis and inter-evaluator as well as inter-laboratory correspondences were analysed [29].

Azithromycin has been found to change the composition of subgingival biofilms when compared to placebo. Oteo et al. [13] observed a statistically significant decrease in the prevalence of *P. micra* and *P. gingivalis* after 1 month and *A. actinomycetemcomitans* after 6 months in patients treated with azithromycin compared to placebo, while minor changes of these parameters were observed in the control group. These results are similar to the results of the present study, which found a statistically significant decrease in the proportion and total counts of *P. gingivalis* as well as the eradication of *A. actinomycetemcomitans* in test subjects. Han et al. [18] only reported a decrease of *F.nucleatum*, while Gomi et al. [33], Yashima et al. [34] and Sampaio et al. [25] reported no changes in the prevalence of red complex bacteria.

Several studies [20] and systematic reviews [24, 35] have reported that the benefit of systemic azithromycin as an adjunct to non-surgical periodontal therapy is greatest in initially deep pockets (> 5 mm), but not at shallow or moderate sites. Contrary to these conclusions, the 6-month results of the present study did not show any significant clinical differences between test and control group subjects. The median PD decreased from 4.1 mm to 2.9 mm in the test group and from 3.9 mm to 2.7 mm in the control group, while a baseline CAL of 4.2 mm in both groups resulted in the same 3.3 mm CAL 6 months post-treatment. Significant differences were not observed at the level of deeper initial pockets (PD > 5 mm) with BOP either, the differences of median gains in PD and CAL being similar in both groups (control group: 2.6 mm and 2.1 mm, respectively; test group: 2.5 mm and 2.2 mm, respectively). The same conclusions were also drawn by Han et al. [18] and Sampaio et al. [25]. Both studies included very advanced periodontitis cases only and reported no significant differences between test and control groups in PD and CAL at sites with initial PD > 6 mm after 6 months, observing improvements in both groups regardless of treatment mode. The lack of intergroup differences in the present study may be attributed to the success rate of the chosen SRP protocol, whereby persisting pockets were re-instrument 3 months after treatment. Namely, our 3-month unpublished results showed a significantly lower number of residual DS and DS on molars in test subjects harbouring *A. actinomycetemcomitans* at baseline, compared to control group subjects harbouring *A. actinomycetemcomitans,* thus indicating that after 3 months, azithromycin does in fact provide significant benefits compared to placebo. This matches the 6-month microbiologic results which show that the eradication of *A. actinomycetemcomitans* was significantly more frequent in subjects who received azithromycin. At the 3-month follow-up visit, however, sites with persisting DS (PD ≥ 5 mm and BOP) were re-instrumented. This is the most likely explanation for the lack of intergroup differences after 6 months, since clinical parameters of both groups further improved between the 3 and 6-month examinations, but more so in control group subjects, resulting in statistically nonsignificant differences in 6-month intergroup clinical variables. We may hypothesize that azithromycin significantly reduces the short-term need for surgical treatment in subjects with *A. actinomycetemcomitans,* but in the long run appears to yield similar results and no important improvements than mechanical treatment alone. The same pattern can be seen in other short-term studies [36], where clinical improvements were observed 3 months post-treatment, but were found to fade in long term studies [37]. A negative relation between the length of follow-up and net change in probing depth was also reported by Zhang et al. [24] in a meta-analysis of 14 trials.

Feres et al. [38] attributed the poor clinical outcomes of azithromycin to its limited effect on the subgingival microflora, reporting that red complex bacteria accounted for 12% of all evaluated species 1 year after treatment, regardless of the protocol used – azithromycin + SRP or placebo + SRP. In contrast to amoxicillin/metronidazole, azithromycin is not bactericidal but bacteriostatic [9, 39]. Yet efficient and long-lasting clinical results, especially with regards to obligate anaerobes, demand a fast and massive decrease in total bacterial load, in addition to recolonization with non-pathogenic bacteria [39]. It is harder to establish this balance using a bacteriostatic antibiotic such as azithromycin [25, 37].

Another reason for the variability of results in azithromycin related trials may be the inclusion criteria, especially the stage of periodontitis itself [5]. Sampaio et al. [25] selected very severe periodontitis patients with an average baseline PD of 5.02 mm as well as frequent suppuration and found no statistically significant differences between the test and control groups 6 months after treatment, while, Oteo et al. [13], on the other hand, included moderate periodontitis patients with an average baseline PD of 2.99 mm and reported significantly lower PDs in the test group. According to this data it is possible to speculate that azithromycin, contrary to general opinion [24], provides beneficial effects only in mild, microbiologically undemanding clinical conditions. Subjects in the present study were diagnosed with moderately severe periodontitis (stage III or IV; average PD = 4.08 mm; average number of sites with PD > 6 mm = 15), showing no intergroup differences in clinical parameters. In addition, around 90% of DS were healed in both groups regardless of treatment type, tooth type (molar/non-molar) and site position (interdental/non-interdental). No significant differences between SRP + placebo and SRP + azithromycin treatment protocols in non-smokers with generalized moderate to advanced chronic periodontitis were likewise found by Saleh et al. [36]. On the contrary, Jentsch et al. [40] found that the use of azithromycin, as an adjunct to SRP, resulted in significantly lower CAL and BOP than even treatment with the gold standard – amoxicillin/metronidazole – and concluded that the administration of azithromycin could be an alternative to the use of amoxicillin/metronidazole in patients with moderate or severe chronic periodontitis. Nevertheless, it is important to note that most studies which found positive effects of azithromycin [33, 34, 40] reported worse outcomes of mechanical treatment (control groups) in comparison to our results and the results of Sampaio et al. [25] or Han et al. [18]. Taken together, due to the problems regarding the indiscriminate use of antibiotics in periodontology (systemic side effects, microbiological adverse effects, bacterial resistances), the potential of mechanical debridement protocols should be exploited to the maximum, perhaps even with additional mechanical instrumentation of sites that show residual PD ≥ 5 mm and BOP after SRP, and the use of systemic antibiotics in periodontitis should be restricted to certain patients and certain periodontal conditions only (e.g., aggressive periodontitis, severe and progressing forms of periodontitis) [6, 41].

An important finding of this study is also the outcome of the multivariant multilevel logistic regression, which indicated a lower odds ratio for the healing of residual sites on molars. This is in line with studies that have shown low success rates when treating molars and frequent extractions of furcation involved teeth that are lost either after non-surgical or surgical treatment [42–46]. In addition, furcation involved teeth often do not allow for optimal instrumentation due to anatomic features and limited access [46].

## Conclusions

To summarize, in comparison to SRP, the systemic use of azithromycin as an adjunct to SRP decreased the proportion and total counts of *P. gingivalis*, decreased the proportion of *C. rectus* and resulted in more frequent eradication of *A. actinomycetemcomitans* and *C. rectus*. Its use showed significant improvements in periodontal parameters and the number of residual diseases sites (sites with PD ≥ 5 mm and BOP) 6-months after treatment, which were, however, equivalent to subjects who were treated with SRP alone. Irrespective of treatment type, 90% of diseased sites were healed in more than half of the subjects. A lower odds ratio was found for the healing of sites on molars. It can therefore be concluded that the empiric use of azithromycin in the treatment of generalized stage III/IV periodontitis is unfounded.

## Supplementary information

**Additional file 1 Supplemental Table 1** Proportions (%) of healed sites (HS) at baseline and after 6 months (Me [IQR]).

**Additional file 2 Supplemental Table 2** Frequency of detection of 9 periodontopathogens in positive samples before and 6 months after treatment (% [n]).

**Additional file 3 Supplemental Table 3** Eradication of bacteria present at baseline, 6 months after treatment (n [%]).

**Additional file 4 Supplemental Table 4** Proportions of 9 periodontopathogens in positive samples before and 6 months after treatment (Me % [IQR]).

**Additional file 5 Supplemental Table 5** Total counts (CFU/ml) of 9 periodontopathogens in positive samples before and 6 months after treatment (Me counts [IQR]).

## Data Availability

The data generated and analysed during the presented study are available from corresponding author on reasonable request. Parts of the present manuscript represent the content of an undergraduate student research project of KP and KČ and thesis of SM.

## Abbreviations

CAL: Clinical attachment loss
CFU: Colony forming unit
DS: Diseased site (site with PD ≥ 5 mm and BOP)
GBI: Dichotomous gingival bleeding index
HS: Healed site
IQU: Interquartile range
Me: Median
OR: Odds ratio
PlI: Dichotomous plaque index
PD: Pocket depth
REC: Gingival recession
SRP: Scaling and root planing
