# Supplementary material for: Influence of adjunctive azithromycin on microbiological and clinical outcomes in periodontitis patients: 6-month results of randomized controlled clinical trial
Source: BMC Oral Health. 2020 Sep 1;20:241. doi: 10.1186/s12903-020-01209-0 (PMC7465355; doi:10.1186/s12903-020-01209-0)
Supplement: Supplementary file 1 — Additional file 1 Supplemental Table 1 Proportions (%) of healed sites (HS) at baseline and after 6 months (Me [IQR]). [file 12903_2020_1209_MOESM1_ESM.docx]

**Supplemental Table 1** Proportions (%) of healed sites (HS) at baseline and after 6 months (Me [IQR])

|  | **Control group** | **Test group** |
| --- | --- | --- |
| **Baseline** |  |  |
| HS | 32.1 (19.2 – 42.9) | 21.8 (14.2 – 42.7) |
| **6-months post-treatment** |  |  |
| HS | 91.3 (73.3 – 97.6) | 89.3 (80.7 – 100.0) |
| HS on molars | 85.7 (69.6 – 100.0) | 90.6 (75.9 – 100.0) |
| HS on non-molars | 93.8 (76.7 – 100.0) | 93.5 (88.5 – 100.0) |
| Interdental HS | 88.2 (72.3 – 98.4) | 90.5 (77.8 – 100.0) |
| Non-interdental HS | 100.0 (71.4 – 100.0) | 97.1 (80.0 – 100.0) |

^Me: median value, IQR: interquartile range, HS, healed sites.^
