# Supplementary material for: Influence of adjunctive azithromycin on microbiological and clinical outcomes in periodontitis patients: 6-month results of randomized controlled clinical trial
Source: BMC Oral Health. 2020 Sep 1;20:241. doi: 10.1186/s12903-020-01209-0 (PMC7465355; doi:10.1186/s12903-020-01209-0)
Supplement: Supplementary file 2 — Additional file 2 Supplemental Table 2 Frequency of detection of 9 periodontopathogens in positive samples before and 6 months after treatment (% [n]). [file 12903_2020_1209_MOESM2_ESM.docx]

**Supplemental Table 2** Frequency of detection of 9 periodontopathogens in positive samples before and 6 months after treatment (% [n])

|  | **Control group** | | | **Test group** | | |
| --- | --- | --- | --- | --- | --- | --- |
|  | Baseline | 6 months | p | Baseline | 6 months | p |
| Aa | 42.1 (8) | 42.1 (8) | 1.000 | 31.6 (6) | 21.1 (4) | 0.625 |
| Pg | 68.4 (13) | 47.4 (9) | 0.219 | 57.9 (11) | 31.6 (6) | 0.125 |
| Pi | 89.5 (17) | 78.9 (15) | 0.500 | 94.7 (18) | 84.2 (16) | 0.500 |
| Tf | 78.9 (15) | 52.6 (10) | 0.063 | 89.5 (17) | 63.2 (12) | 0.180 |
| Pm | 89.5 (17) | 94.7 (18) | 1.000 | 100.0 (19) | 78.9 (15) | 0.134 |
| Fn | 63.2 (12) | 73.7 (14) | 0.625 | 63.2 (12) | 68.4 (13) | 1.000 |
| Cr | 63.2 (12) | 26.3 (5) | 0.039* | 42.1 (8) | 0.0 (0) | 0.013* |
| Ec | 5.3 (1) | 10.5 (2) | 1.000 | 0.0 (0) | 10.5 (2) | 0.479 |
| Co | 0.0 (0) | 5.3 (1) | 1.000 | 0.0 (0) | 10.5 (2) | 0.479 |

^Aa, Aggregatibacter actinomycetemcomitans – Pg, Porphyromonas gingivalis – Pi, Prevotella intermedia – Ec, Eikenella corrodens – Fn, Fusobacterium nucleatum – Pm, Parvimonas micra – Cr, Campylobacter rectus – Co, Capnocytophaga ochracea – Tf, Tannerella forsythia – *, statistically significant change in comparison to baseline.^
