# Supplementary material for: Influence of adjunctive azithromycin on microbiological and clinical outcomes in periodontitis patients: 6-month results of randomized controlled clinical trial
Source: BMC Oral Health. 2020 Sep 1;20:241. doi: 10.1186/s12903-020-01209-0 (PMC7465355; doi:10.1186/s12903-020-01209-0)
Supplement: Supplementary file 3 — Additional file 3 Supplemental Table 3 Eradication of bacteria present at baseline, 6 months after treatment (n [%]). [file 12903_2020_1209_MOESM3_ESM.docx]

**Supplemental Table 3** Eradication of bacteria present at baseline, 6 months after treatment (n [%])

|  | **Control group** | **Test group** | **p** |
| --- | --- | --- | --- |
| Aa | 0 (0.0) | 3 (50.0) | 0.013* |
| Pg | 5 (38.5) | 6 (54.5) | 0.430 |
| Pi | 2 (11.8) | 2 (11.1) | 0.952 |
| Tf | 5 (33.3) | 7 (41.2) | 0.647 |
| Pm | 1 (5.9) | 4 (21.1) | 0.174 |
| Fn | 1 (8.3) | 4 (33.3) | 0.121 |
| Cr | 8 (66.7) | 8 (100.0) | 0.029* |
| Ec | 1 (100.0) | / | / |
| Co | / | / | / |

^Aa, Aggregatibacter actinomycetemcomitans – Pg, Porphyromonas gingivalis – Pi, Prevotella intermedia – Ec, Eikenella corrodens – Fn, Fusobacterium nucleatum – Pm, Parvimonas micra – Cr, Campylobacter rectus – Co, Capnocytophaga ochracea – Tf, Tannerella forsythia – *, statistically significant change.^
