# Supplementary material for: Influence of adjunctive azithromycin on microbiological and clinical outcomes in periodontitis patients: 6-month results of randomized controlled clinical trial
Source: BMC Oral Health. 2020 Sep 1;20:241. doi: 10.1186/s12903-020-01209-0 (PMC7465355; doi:10.1186/s12903-020-01209-0)
Supplement: Supplementary file 4 — Additional file 4 Supplemental Table 4 Proportions of 9 periodontopathogens in positive samples before and 6 months after treatment (Me % [IQR]). [file 12903_2020_1209_MOESM4_ESM.docx]

**Supplemental Table 4** Proportions of 9 periodontopathogens in positive samples before and 6 months after treatment (Me % [IQR])

|  | **Placebo group** | | | **Test group** | | |
| --- | --- | --- | --- | --- | --- | --- |
|  | Baseline | 6 months | p | Baseline | 6 months | p |
| Aa | 0.00 (0.00– 0.17) | 0.00 (0.00–0.18) | 0.398 | 0.00 (0.00–0.17) | 0.00 (0.00–0.00) | 0.866 |
| Pg | 11.01 (0.00–31.5) | 0.00 (0.00–26.06) | 0.470 | 4.85 (0.00–20.16) | 0.00 (0.00–2.20) | 0.006* |
| Pi | 3.67 (2.02–11.39) | 4.17 (0.57–10.62) | 0.407 | 2.58 (0.91–9.63) | 1.77 (0.26–7.32) | 0.149 |
| Tf | 4.65 (2.25–7.53) | 0.67 (0.00–4.40) | 0.008* | 6.15 (2.94–9.55) | 1.46 (0.00–2.91) | 0.001* |
| Pm | 3.00 (1.19–5.78) | 3.85 (1.75–5.18) | 0.212 | 3.85 (1.90–5.94) | 3.88 (1.10–6.72) | 0.904 |
| Fn | 0.42 (0.00–2.58) | 1.05 (0.25–2.79) | 0.650 | 0.86 (0.00–2.51) | 1.26 (0.00–2.02) | 0.492 |
| Cr | 1.30 (0.00–2.45) | 0.00 (0.00–0.34) | 0.196 | 0.00 (0.00–1.12) | 0.00 (0.00–0.00) | 0.012* |
| Ec | 0.00 (0.00–0.00) | 0.00 (0.00–0.00) | 1.000 | 0.00 (0.00–0.00) | 0.00 (0.00–0.00) | 0.180 |
| Co | 0.00 (0.00–0.00) | 0.00 (0.00–0.00) | 0.317 | 0.00 (0.00–0.00) | 0.00 (0.00–0.00) | 0.180 |

^Me: median value, IQR: interquartile range , Aa, Aggregatibacter actinomycetemcomitans – Pg, Porphyromonas gingivalis – Pi, Prevotella intermedia – Ec, Eikenella corrodens – Fn, Fusobacterium nucleatum – Pm, Parvimonas micra – Cr, Campylobacter rectus – Co, Capnocytophaga ochracea – Tf, Tannerella forsythia – *, statistically significant change in comparison to baseline.^
