# Supplementary material for: Influence of adjunctive azithromycin on microbiological and clinical outcomes in periodontitis patients: 6-month results of randomized controlled clinical trial
Source: BMC Oral Health. 2020 Sep 1;20:241. doi: 10.1186/s12903-020-01209-0 (PMC7465355; doi:10.1186/s12903-020-01209-0)
Supplement: Supplementary file 5 — Additional file 5 Supplemental Table 5 Total counts (CFU/ml) of 9 periodontopathogens in positive samples before and 6 months after treatment (Me counts [IQR]). [file 12903_2020_1209_MOESM5_ESM.docx]

**Supplemental Table 5** Total counts (CFU/ml) of 9 periodontopathogens in positive samples before and 6 months after treatment (Me counts [IQR])

|  | **Placebo group (n = 19)** | | | **Test group (n = 19)** | | |
| --- | --- | --- | --- | --- | --- | --- |
|  | Baseline | 6 months | p | Baseline | 6 months | p |
| Aa | 0 (0 – 15,892) | 0 (0 – 5,435) | 0.779 | 0 (0 – 12,922) | 0 (0 – 0) | 0.866 |
| Pg | 1,452,660 (0 – 3,135,313) | 0 (0 – 1,913,894) | 0.124 | 330,046 (0 – 2,244,316) | 0 (0 – 56,115) | 0.003* |
| Pi | 593,439 (132,132 – 1,384,977) | 65,997 (26,391 – 280,377) | 0.031* | 329,967 (118,715 – 1,092,062) | 131,881 (10,044 – 297,033) | 0.050* |
| Tf | 528,000 (207,675 – 858,115) | 19,819 (0,0 – 257,380) | 0.008* | 660,607 (237,303 – 1,418,472) | 39,507 (0 – 158,730) | 0.001* |
| Pm | 396,000 (69,300 – 759,429) | 118,918 (39,161 – 264,099) | 0.084 | 330,422 (197,897 – 1,055,485) | 330,132 (36,297 – 627,247) | 0.748 |
| Fn | 65,577 (0 – 197,505) | 39,600 (6,593 – 115,572) | 0.233 | 72,669 (0 – 198,066) | 19,875 (0 – 125,318) | 0.381 |
| Cr | 66,784 (0 – 263,437) | 0 (0 – 3,295) | 0.033* | 0 (0 – 175,144) | 0 (0 - 0) | 0.012* |
| Ec | 0 (0 – 0) | 0 (0 – 0) | 1.000 | 0 (0 – 0) | 0 (0 – 0) | 0.180 |
| Co | 0 (0 – 0) | 0 (0 – 0) | 0.317 | 0 (0 - 0) | 0 (0 - 0) | 0.180 |

^Me: median value, IQR: interquartile range, Aa, Aggregatibacter actinomycetemcomitans – Pg, Porphyromonas gingivalis – Pi, Prevotella intermedia – Ec, Eikenella corrodens – Fn, Fusobacterium nucleatum – Pm, Parvimonas micra – Cr, Campylobacter rectus – Co, Capnocytophaga ochracea – Tf, Tannerella forsythia – *, statistically significant change in comparison to baseline.^
